# Supplementary material for: Machine learning-based development of Gadolinium binding peptides
Source: bioRxiv. 2025 Dec 16:2025.12.14.694215. Preprint. [Version 1] doi: 10.64898/2025.12.14.694215 (PMC12746035; doi:10.64898/2025.12.14.694215)
Supplement: Supplement 1 [file media-1.pdf]

Machine learning-based development of Gadolinium binding peptides

Nir A. Dayan[1], Makayla Long[2], Nicolas Scalzitti[1,3], Iliya Miralavy[3], Daniel Holmes[4], Mark Kocherovsky[3], Wolfgang Banzhaf[3]*, and Assaf A. Gilad[1,5,6]*

[1] Department of Chemical Engineering & Material Science, Michigan State University, East Lansing, Michigan 48824, USA
[2] Michigan State University College of Human Medicine, Grand Rapids, Michigan 49503, USA
[3]Department of Computer Science and Engineering, Michigan state university, 48824 East Lansing, Michigan 48824, USA
[4]Department of Chemistry, Michigan State University, East Lansing, Michigan 48824, USA
[5]Department of Radiology, Michigan State University, East Lansing, Michigan 48824, USA
[6]The Scojen Institute for Synthetic Biology, Reichman University, Herzliya, Israel

*Corresponding authors:

assaf.a.gilad@gmail.com

banzhafw@msu.edu

Electron spin resonance measurements

Electron spin resonance (ESR) spectroscopy[1] was used to assess the relative contributions of bound and free Gd^{3+} ions in solution. EF3 peptide (740 μM) was incubated with $GdCl_3$ under two conditions: (i) 1 mM $GdCl_3$ followed by dialysis to remove unbound metal and (ii) 250 μM $GdCl_3$ without dialysis. Measurements were performed in 50 mM HEPES at 130 K on an X-band continuous-wave ESR spectrometer. Figure S1 shows the comparison of these spectra. The black trace represents EF3 incubated with Gd^{3+} and subsequently dialyzed to remove free ions, while the blue trace corresponds to the spectrum subtraction of (undialyzed EF3 incubated with Gd) minus (free Gd^{3+} spectrum of the same concentration). Thus, the figure does not display the raw undialyzed EF3 or free Gd^{3+} spectra individually, but rather the difference spectrum isolating the contribution of Gd^{3+} bound to the peptide. The dialyzed sample (black trace) exhibits a broad, downfield shifted low-intensity feature characteristic of peptide-bound Gd^{3+}, whose restricted tumbling and altered electronic environment broaden and shift the signal. A small central peak remains, reflecting a minor amount of free Gd^{3+} that persists after dialysis.

The subtracted spectrum (blue trace) retains both a strong central peak and a downfield shoulder. The intense central peak results from incomplete cancellation of the free-Gd transition, which can differ in amplitude or linewidth between samples and therefore does not fully subtract. The downfield shoulder arises from peptide-bound or partially bound Gd^{3+} in the undialyzed mixture. This bound population is not identical to the dialyzed species; it is more heterogeneous, influenced by higher free-Gd concentration and dynamic exchange with bulk solvent, leading to a slightly different ESR position and shape. Together, these data confirm that dialysis removes

most unchelated Gd^{3+} and isolates a stable peptide-bound population, validating the conditions used for subsequent relaxivity measurements.

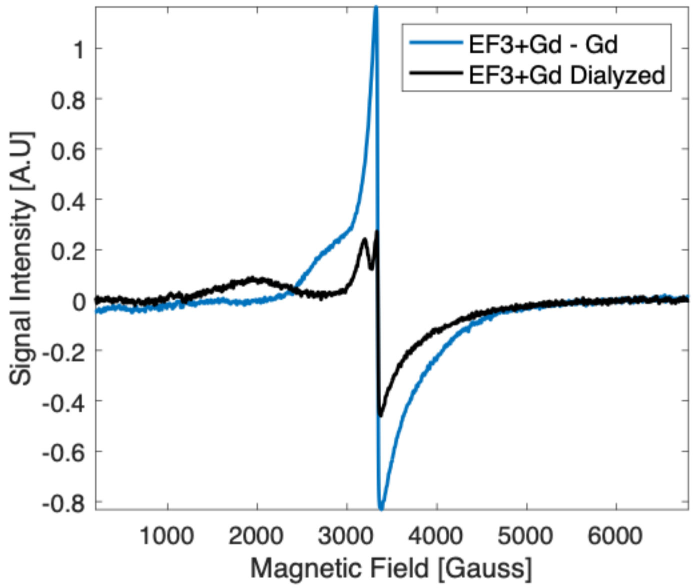

Figure S1. Electron spin resonance (ESR) spectra of EF 3 hand motif (EF3) peptide incubated with Gd^{3+} under two conditions. The black trace represents EF3 (740 µM) incubated with 1 mM $GdCl_3$ and dialyzed to remove unbound metal. The blue trace shows the spectrum subtraction of EF3 (740 µM) incubated with 250 µM $GdCl_3$ without dialysis and free Gd^{3+} of the same concentration. Measurements were performed in 50 mM HEPES at 130 K using X-band continuous-wave ESR. The dialyzed sample exhibits lower intensity and broader lines, consistent with peptide-bound Gd^{3+} and removal of unchelated ions.

Peptide dataset and results

Table S1 contains the entire dataset, peptides 1 to 74 were used as the initial training for POET predictions, dubbed Epoch 1. Epoch 2's training was comprised of the initial training set, Epoch 1, the reversed sequence of EF hands 3 and 4, and additional EF hands (peptides 97-100 in Table S1). The reversed sequences of 2 EF hand motifs were measured to check if POET can consider the entire dataset's reverse sequence as another dataset, this way doubling the amount of

available data. However, MRI results showed that the regular and reversed sequences had different r_1 ratios. Figure S2 provides a graphic representation of the results.

Table S1. Complete list of peptides

	Sample	Sequence	r_1 ratio
**1**	EF hand 1[2]	DPDKDGTIDLKE	1.51
**2**	EF hand 2[2]	DPDKDGTLDAKE	1.52
**3**	EF hand 3[2]	DPDNDGTLDKKE	1.86
**4**	EF hand 4[2]	NPDNDGTIDARE	1.74
**5**	Lanthanide binding[3]	DKDGDTIDEREI	1.31
**6**	CaBM 2[4]	DKDGDGTITTKE	1.29
**7**	CaBM[5]	DKDGNGYISAAE	0.93
**8**	Training	DAPSFAKVVPNR	0.39
**9**	Training	KVNFNKAVSNLK	0.40
**10**	Training	LVKPMNKRWVRM	0.40
**11**	Training	LSNRRGREQYAG	1.01
**12**	Training	GIFKTTKCKHNS	0.86
**13**	Training	KVIRYVVAPMKL	0.86
**14**	Training	CHLKDLRKMGLR	0.99
**15**	Training	VKKYVTPGAPVY	0.98
**16**	Training	QPRMWVNVYTCY	0.97
**17**	Training	NQHTPPKPLNNA	0.97

**18**	Training	LHKDRRKPLPLP	0.92
**19**	Training	QLGVHRNPRWNQ	1.01
**20**	Training	HLRLNKNRMQKS	1.01
**21**	Training	NSSNHSNNMPCQ	1.09
**22**	Training	HQWHRHRPPIRR	0.96
**23**	Training	RWHHVNVLWQDR	0.98
**24**	Training	DNPHQLLPVNWR	0.89
**25**	Training	WFGLQRHLKKKD	0.94
**26**	Training	GQRWLYKMKDSM	0.94
**27**	Training	LDHTWGKWGHQS	0.95
**28**	Training	PVARKVVQICHP	0.98
**29**	Training	DPMRVRPWYVAR	1.00
**30**	Training	PGGVPFRLKMDP	1.01
**31**	Training	VCNRIEPLKPIL	0.95
**32**	Training	GNKKNWRWYKNR	0.93
**33**	Training	KPWHGCASRTKR	0.93
**34**	Training	NKAKACNGMKPR	0.92
**35**	Training	TIKPRMVKLPSV	1.08
**36**	Training	MKVAAAMAPKQV	0.94
**37**	Training	NWRDCLSLIVPN	0.96
**38**	Training	PLNIAISNSPDS	0.51
**39**	Training	DCLAVPPNAVTI	0.95

**40**	Training	VNSDPSNGQMRD	1.22
**41**	Training	RGKMPLRWMTRK	0.96
**42**	Training	IKGMNIKMPTDQ	0.91
**43**	Training	PEGVSWRVLKAN	0.94
**44**	Training	RPPMLNVVRVVG	0.92
**45**	Training	YEYEYEYEYEYE	0.35
**46**	Training	KSKSKSKSKSKS	0.99
**47**	Training	PKVVKDMPPWDT	1.24
**48**	Training	MLMKVPVIRRVW	0.98
**49**	Training	VARVDIDLQAIA	0.94
**50**	Training	VITNNWALAQVR	0.96
**51**	Training	TISDIVKVVIRS	0.96
**52**	Training	KSSKSSKSSKSS	0.95
**53**	Training	WLLDLRRQMDDT	1.45
**54**	Training	RDPDKNNRDWPI	1.06
**55**	Training	LDDDTPDRNRDW	1.74
**56**	Training	VGQNNDERRRQR	1.01
**57**	Training	PGPPAGPTLSNR	1.02
**58**	Training	NSPQLNLNPPQS	0.99
**59**	Training	NRSMPLPMNPGD	1.16
**60**	Training	NAARMNRLNDAM	1.04
**61**	Training	ANNNPWSGMMNG	0.96

**62**	Training	MWVKGMKHKKMK	1.01
**63**	Training	VPKDKRKKLTNP	0.95
**64**	Training	AACPNLAVAAPM	0.94
**65**	Training	VPKRLVVVMNTC	1.06
**66**	Training	EAPMPKVNVIVN	1.05
**67**	Training	PLPDNVVKAVVW	1.02
**68**	Training	CKPARTDWPPMP	1.01
**69**	Training	CATNAKNNNMRG	1.00
**70**	Training	VINKVISNPCVN	0.99
**71**	Training	LHSQWLKVDHLL	1.01
**72**	Training	QTATENSQMNSG	1.14
**73**	Training	KKKKKKKKKKKK	1.01
**74**	Training	KSSSKSSSKSSS	0.96
**75**	POET Motif (Epoch 1)	MDDLTGDNKNWV	1.44
**76**	POET Motif (Epoch 1)	QDTTMMTRLDTM	1.06
**77**	POET Motif (Epoch 1)	VDRNTDTDSVDT	1.98
**78**	POET Motif (Epoch 1)	KVDCDDVDRLDS	2.00
**79**	POET Motif (Epoch 1)	DMMHDKTDCNDT	1.71
**80**	POET Motif (Epoch 1)	TCTPTNTLNTMD	1.23
**81**	POET Motif (Epoch 1)	IDMTCSMTDDDT	1.00
**82**	POET Motif (Epoch 1)	QTCRNDGTIDKH	1.10
**83**	POET Motif (Epoch 1)	TNAGTMCTTNTD	1.17

**84**	POET Motif (Epoch 1)	CNDDTAGECTND	2.08
**85**	POET regex (Epoch 1)	CDVDPITCDNDD	1.95
**86**	POET regex (Epoch 1)	LDNPQEDADDDG	2.12
**87**	POET regex (Epoch 1)	LEDDIDCDNNDF	1.77
**88**	POET regex (Epoch 1)	DTMTLNDQETDD	2.05
**89**	POET regex (Epoch 1)	WHCDQTDDIDGH	2.04
**90**	POET regex (Epoch 1)	RDDETLDTLDSG	2.05
**91**	POET regex (Epoch 1)	NSAMADQADDDP	1.12
**92**	POET regex (Epoch 1)	IDCRGTEDDDPN	2.30
**93**	POET regex (Epoch 1)	RVDMDDTIDGMH	1.83
**94**	POET regex (Epoch 1)	NDGGDPMADNDN	1.92
**95**	EF hand 3 reveresed[2]	EKKDLTGDNDPD	1.98
**96**	EF hand 4 reveresed[2]	ERADITGDNDPN	2.08
**97**	Hans EF hand 1[6]	NKDNDDSLEIAE	1.62
**98**	Hans EF hand 2[6]	NPDGDTTLESGE	1.64
**99**	Hans EF hand 3[6]	NKDGDQTLEMDE	1.77
**100**	Hans EF hans 4[6]	DANKDGKLTAAE	1.14
**101**	POET Motif (Epoch 2)	DEDDDLECVDDA	2.02
**102**	POET Motif (Epoch 2)	GDLVDCDEDCDD	1.76
**103**	POET Motif (Epoch 2)	CDEDDEGVKDDD	2.15
**104**	POET Motif (Epoch 2)	NDGDGPCCEDDD	2.07

**105**	POET Motif (Epoch 2)	DDEDDTSGGDTK	2.11
**106**	POET Motif (Epoch 2)	GDDEDDQCHQDG	2.17
**107**	POET Motif (Epoch 2)	EDGDVIGSGKDD	2.07
**108**	POET Motif (Epoch 2)	TEGGTDDTGGND	2.08
**109**	POET Motif (Epoch 2)	CPTDDGHDDHDD	2.10
**110**	POET Motif (Epoch 2)	HDDGVGVDGGDG	1.88
**111**	POET regex (Epoch 2)	CDVDPITCDNDD	2.14
**112**	POET regex (Epoch 2)	SDDNHSDLGDDL	2.12
**113**	POET regex (Epoch 2)	FEDHSDDTDEDT	2.07
**114**	POET regex (Epoch 2)	VDDSDPDDVKDT	2.10
115	POET regex (Epoch 2)	DDDSDEDSTDSH	1.51
116	POET regex (Epoch 2)	DSDEDTHVDCQE	2.16
117	POET regex (Epoch 2)	TQDSDDGMEDED	2.09
118	POET regex (Epoch 2)	STDTAGDDATEG	1.05
119	POET regex (Epoch 2)	ENHSGQHCHDTG	1.81
120	POET regex (Epoch 2)	VCEDHDPNDSHI	1.88

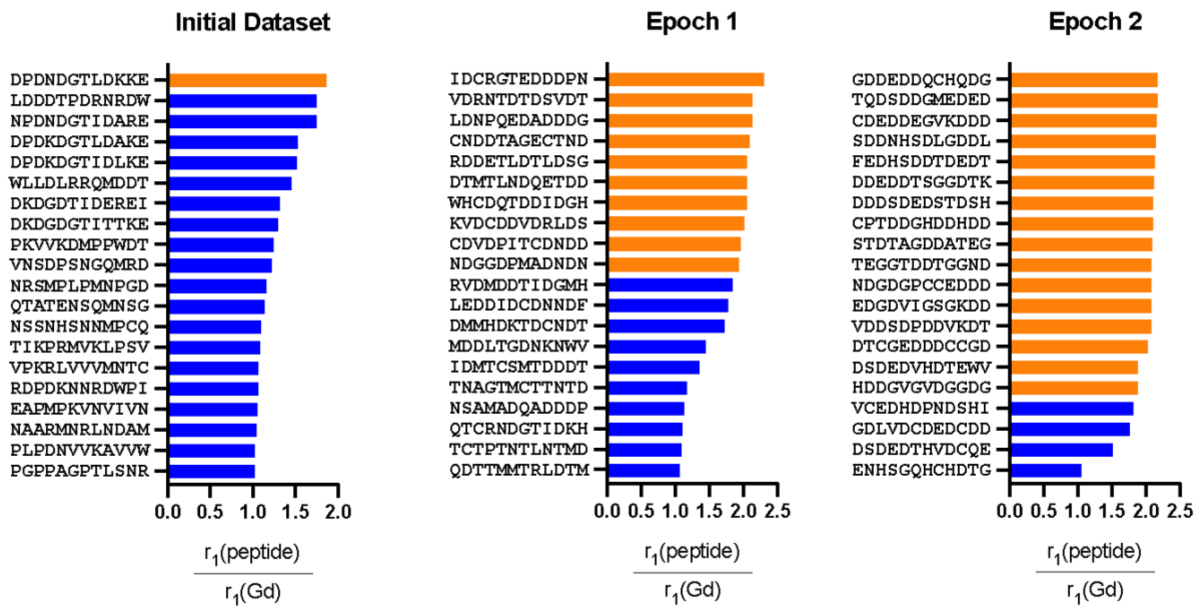

Figure S2. The higher the r_1 ratio, the better the MRI contrast. $GdCl_3$ is used as an internal control. Left - Partial initial dataset for POET. Middle and right – POET predictions from the initial dataset. Orange bars have an r_1 ratio equal to or higher than EF hand 3, the best peptide in the initial dataset.

REFERENCES

1. Jeschke G. Electron Paramagnetic Resonance Spectroscopy. *Ullmann's Encyclopedia of Industrial Chemistry*. 2013.

2. Gutenthaler SM, Tsushima S, Steudtner R, et al. Lanmodulin peptides - unravelling the binding of the EF-Hand loop sequences stripped from the structural corset. *Inorg Chem Front*. Aug 09 2022;9(16):4009-4021. doi:10.1039/d2qi00933a

3. Welch JT, Kearney WR, Franklin SJ. Lanthanide-binding helix-turn-helix peptides: Solution structure of a designed metallonuclease. *Proceedings of the National Academy of Sciences*. 2023/05/16 2003;100(7):3725-3730. doi:10.1073/pnas.0536562100

4. Sharma Y, Chandani S, Sukhaswami MB, Uma L, Balasubramanian D, Fairwell T. Modified helix-loop-helix motifs of calmodulin--The influence of the exchange of helical regions on calcium-binding affinity. *Eur J Biochem*. Jan 15 1997;243(1-2):42-8. doi:10.1111/j.1432-1033.1997.0042a.x

5. Wójcik J, Góral J, Pawłowski K, Bierzyński A. Isolated calcium-binding loops of EF-hand proteins can dimerize to form a native-like structure. *Biochemistry*. Jan 28 1997;36(4):680-7. doi:10.1021/bi961821c

6. Mattocks JA, Jung JJ, Lin CY, et al. Enhanced rare-earth separation with a metal-sensitive lanmodulin dimer. *Nature*. Jun 2023;618(7963):87-93. doi:10.1038/s41586-023-05945-5
